# Seismic response analysis of loess site under far-field bedrock ground motion of the Wenchuan earthquake

**DOI:** 10.1371/journal.pone.0254871

**Published:** 2021-07-30

**Authors:** Tuo Chen

**Affiliations:** State Key Laboratory for GeoMechanics and Deep Underground Engineering, School of Mechanics and Civil Engineering, China University of Mining and Technology, Xuzhou, Jiangsu, China; University of Genova, ITALY

## Abstract

In this paper, considering the far-field seismic input, an accelerogram recorded in the bedrock at Wuquan Mountain in Lanzhou city during the 2008 Wenchuan Ms8.0 earthquake was selected, and numerical dynamic analyses were conducted. The one-dimensional equivalent linear method was implemented to estimate the ground motion effects in the loess regions. Thereafter, slope topographic effects on ground motion were studied by applying the dynamic finite-element method. The results revealed the relationship between the PGA amplification coefficients and the soil layer thickness, which confirmed that the dynamic response of the sites had obvious nonlinear characteristics. The results also showed that there was an obvious difference in the dynamic magnification factor between the short-period and long-period structures. Moreover, it was found that the amplification coefficient of the observation point at the free surface was greater than the point inside the soil at the same depth, which mainly occurred in the upper slope. Through this study, the quantitative assessment of ground motion effects in loess regions can be approximately estimated, and the amplification mechanism of the far-field ground motion mechanism can be further explained. In addition to the refraction and reflection theory of seismic waves, the resonance phenomenon may help explain the slope topographic effect through spectrum analysis.

## Introduction

Loess and loessic deposits account for approximately 6.63% of the total area of China. Loess thickness generally exceeds 100 m over large areas of the Loess Plateau, with a maximum recorded thickness of 335 m in eastern Gansu Province near the city of Lanzhou [1]. Loess has a highly porous structure and weakly cemented characteristics, which show a strong dynamic catastrophic characteristic and highly dynamic vulnerability. Together with the impressive features of geomorphology and deposit thickness, seismic damage becomes more serious in the loess areas than in nonloessic areas [2].

Based on the conventional recognition that the disasters caused by near-field earthquakes are more obvious than those caused by far-field earthquakes, the study of near-field ground motion has attracted more attention, whereas the seismic disasters in far-field areas caused by strong earthquakes should not be neglected [3–6]. First, ground motion records have been used by some researchers to investigate the characteristics of far-field ground motion [7–10]. They confirmed that seismic ground motion changes noticeably because of the filtering effect and amplification of the thick soil layer, and part of the long-period ground motion may be greatly enhanced, which may cause severe damage to high-rise buildings. Furthermore, extensive research on site effects has been conducted using different numerical calculation methods. Arslan [11] confirmed the influence of the nonlinearity of soil behavior and elucidated the effect of nonlinearity on the site response. Hartzell [12] studied the influence of topography on ground motion and evaluated topographic amplification utilizing a variety of methods. Pandey [13] discussed the influence of local site conditions on the characteristics of strong ground motion records, and their further effects on hazard studies have been investigated. Tran et al. [14] discussed the influence of random variations in soil characterization on seismic site response. Glinsky [15] discussed topographic and geological site effects based on a discontinuous Galerkin finite element method. However, seismic responses and site effects in loess areas have not been systematically studied. Only a few researchers at the Lanzhou Institute of Seismology in Gansu Province have conducted related studies [16–18]. The amplification effects and slope effects have been confirmed at loess sites, while the quantitative assessment of ground motion effects should be further investigated, especially at different loess thicknesses and slope angles. Moreover, the calculated results need to be calibrated by the basic methodology.

In this study, the far-field accelerogram recorded in the bedrock at Wuquan Mountain in Lanzhou city during the 2008 Wenchuan Ms8.0 earthquake was used as the seismic input in the numerical analysis. Then, the one-dimensional equivalent linear method was utilized to evaluate the ground motion effects in the loess regions under far-field earthquake excitations. Moreover, slope topographic effects on ground motion were studied by applying the dynamic finite element method. Through the comparison and analysis of the numerical results of applying different methods, the quantitative assessment of ground motion effects in mountainous areas in southeastern Gansu Province can be approximately estimated, and the amplification mechanism of the far-field ground motion mechanism can be further explained. The results of this research will be of interest for researchers and civil engineers and will provide some guidance for resisting earthquakes and preventing disasters in loess areas in China.

## Far-field bedrock ground motion

The distance from an earthquake source (epicenter and fault) is used to differentiate between the near field and far field. The near field is identified in earthquake engineering when this distance lies in the range from 20 to 60 km, whereas the far field refers to a distance greater than 200 km [19]. Moreover, there exists some difference in the spectrum characteristics between the near-field and far-field ground motions. Near-field ground motions consist of a major portion of the fault energy in the form of pulses, which tend to have a maximum Fourier spectrum in limited periods, whereas far-field ground motions have a maximum Fourier spectrum in a broad range of periods [20].

**Fig 1:**
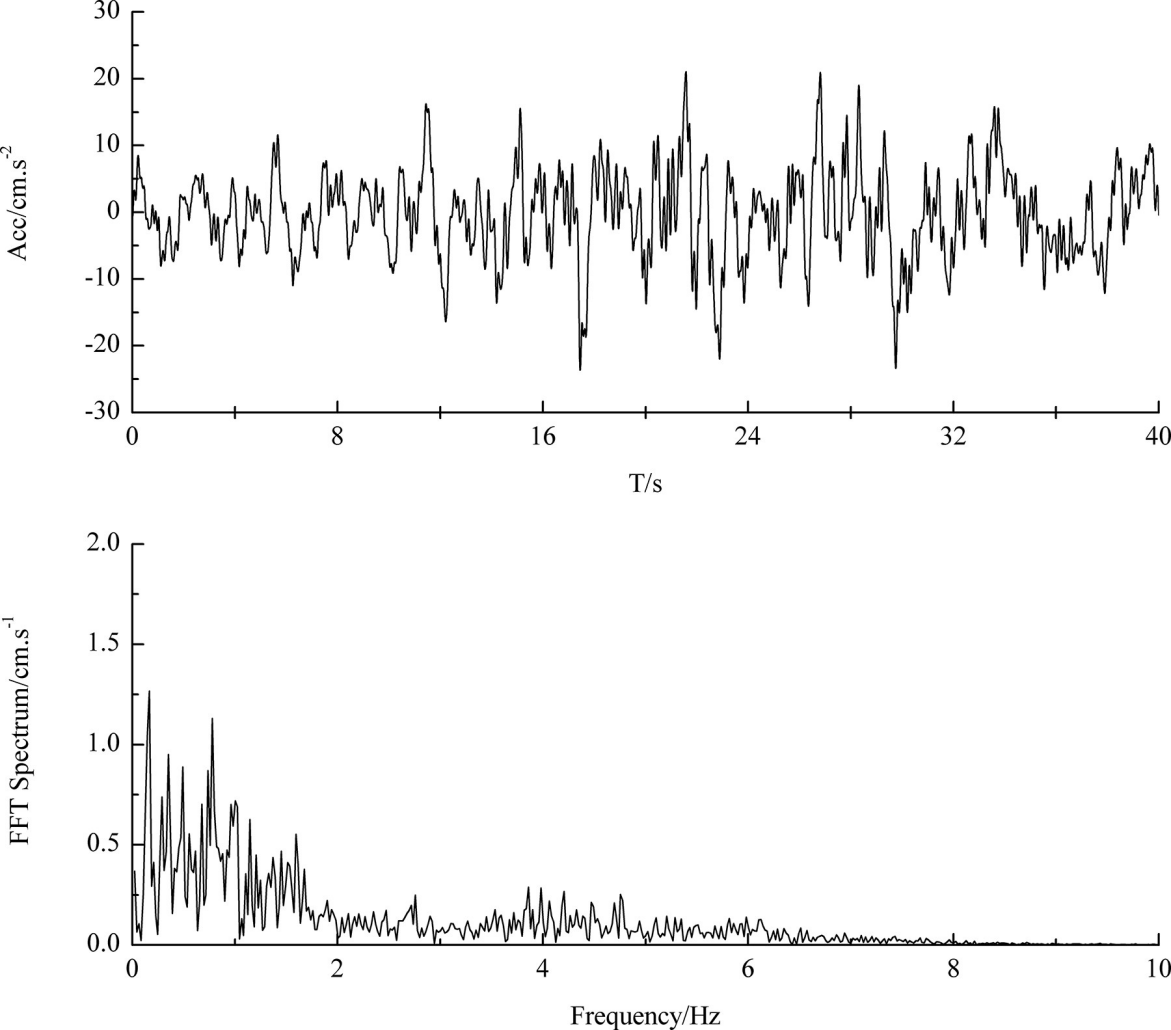
Horizontal acceleration history at Wuquan mountain during Wenchuan earthquake.

In this paper, far-field earthquake action was considered and the far-field earthquake accelerogram recorded in the bedrock at Wuquan Mountain in Lanzhou city during the 2008 Wenchuan Ms8.0 earthquake was used as the dynamic input seismic motion. Fig 1 illustrates the horizontal acceleration time history of this far-field bedrock ground motion. The maximum acceleration of the input motion was 23 cm.s^-2^, and the energy of earthquake ground motion was concentrated in the range of 0.1–2 Hz.

### Seismic ground motion analysis

#### Calculation model and parameters

Site response analysis is crucial for defining seismic hazards and the distribution of damage during earthquakes. In this paper, the one-dimensional equivalent linear method was used to perform seismic ground motion analysis [21]. This method is implemented based on elastic wave propagation theory, which assumes that the response of soil deposits is predominantly caused by the vertical propagation of shear waves from the bedrock. The wave equation in a linear viscoelastic medium can be expressed as:

$$\rho \frac{\partial^{2}u}{\partial t^{2}}=G\frac{\partial^{2}u}{\partial z^{2}}+C\frac{\partial^{3}u}{\partial t\partial z^{2}}$$ (1)

where G is the coefficient of elasticity and C is the viscosity coefficient.

**Fig 2:**
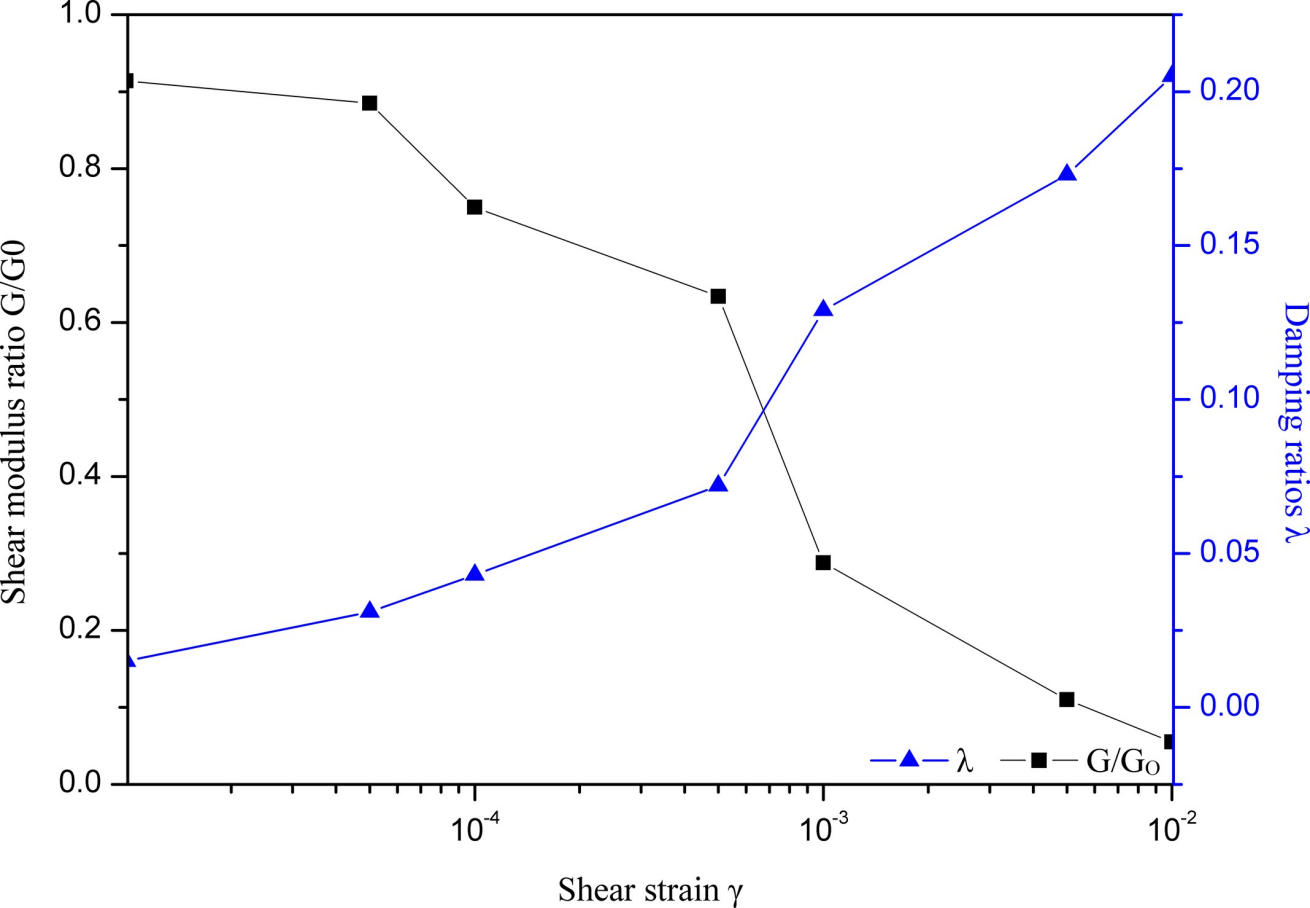
The strain dependent shear modulus ratio and damping ratio for loess.

In this paper, an equivalent nonlinear viscoelastic model was used to solve this equation, and the equivalent shear modulus and damping ratios were used to describe the nonlinear hysteretic properties of the soil material under seismic loading. The strain-dependent shear modulus ratio G/Gmax~γ and damping ratio λ ~γ were revealed by soil dynamic triaxial tests conducted by the Key Laboratory of Loess Earthquake Engineering, China Earthquake Administration. The kinetic parameters of typical loess are shown in Fig 2.

**Fig 3:**
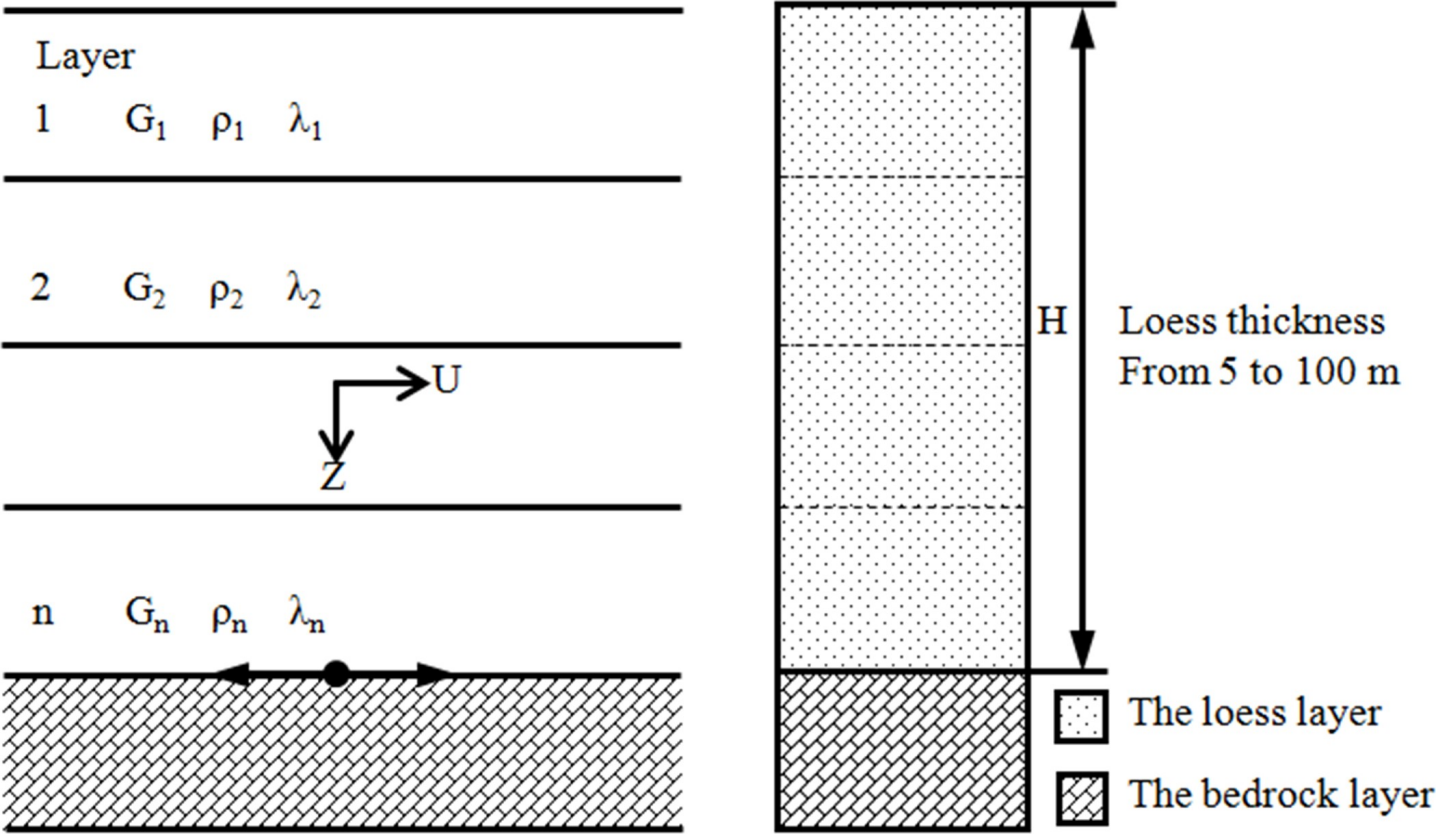
One-dimensional idealization calculation model.

A numerical scheme is presented to compute the seismic response of the loess site. As shown in Fig 3, this calculation model contained two layers: the upper loess layer and bottom bedrock layer. The thickness of the upper loess layer was set to be a variable quantity *H*, which ranged from 5 to 100 m; the thickness of the bottom bedrock layer was also invariant. The calculation was conducted at an interval of 5 m thickness of the loess layer.

It was assumed that the loess soils at various depths have the same shear modulus and damping ratios but different densities and shear wave velocities. According to the in situ shear-wave velocity test results, the shear wave velocity of the soil layer at different depths was determined. The in situ shear-wave velocity tests were conducted by Wang and Wu in Dazhai village [22]. This area is located in the mountainous areas of southeastern Gansu Province, where the thickness of the loess deposit is more than 90 m.

### Numerical results and analysis

**Fig 4:**
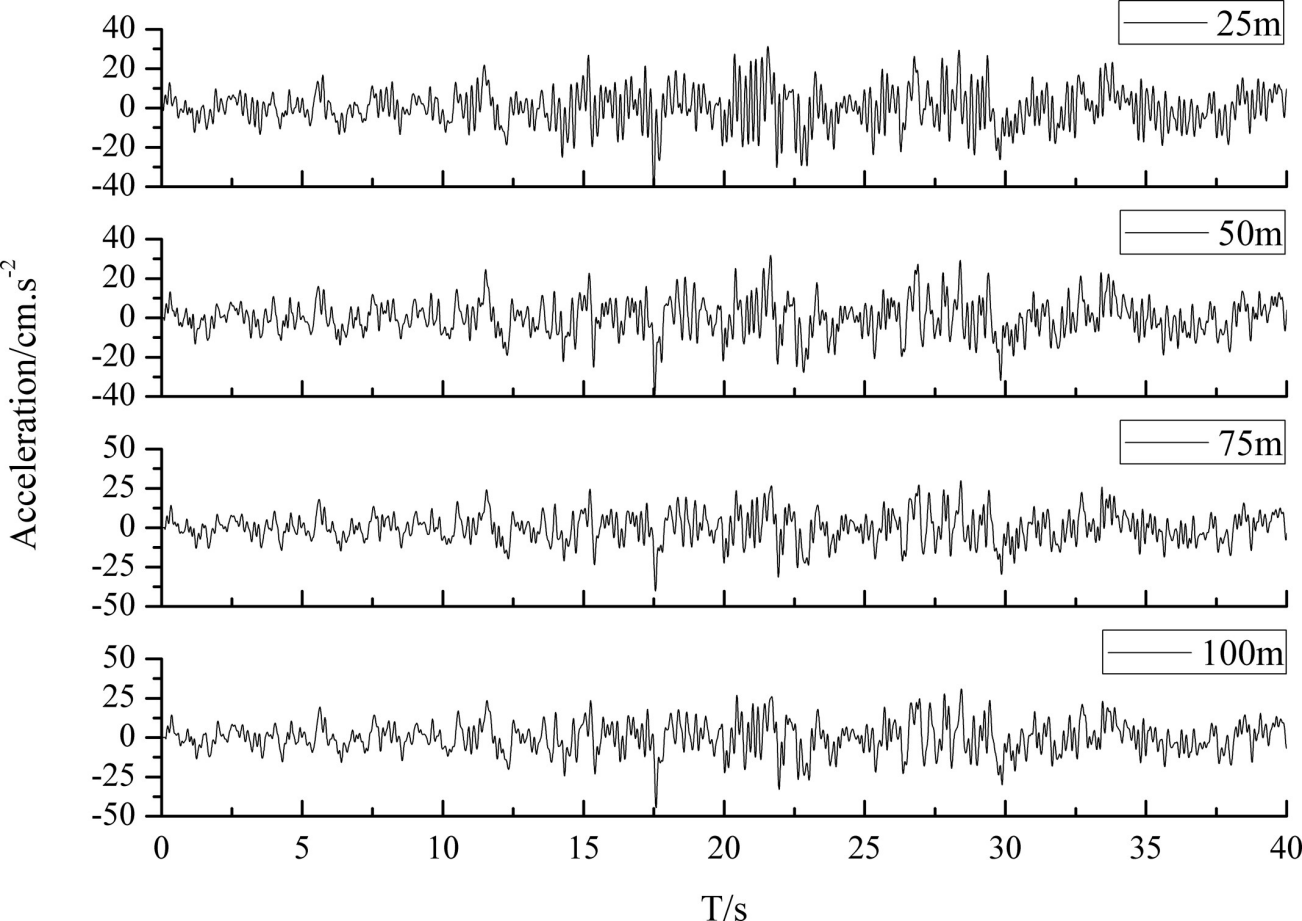
Ground motion acceleration time history of the sites with different overburden thickness.

**Fig 5:**
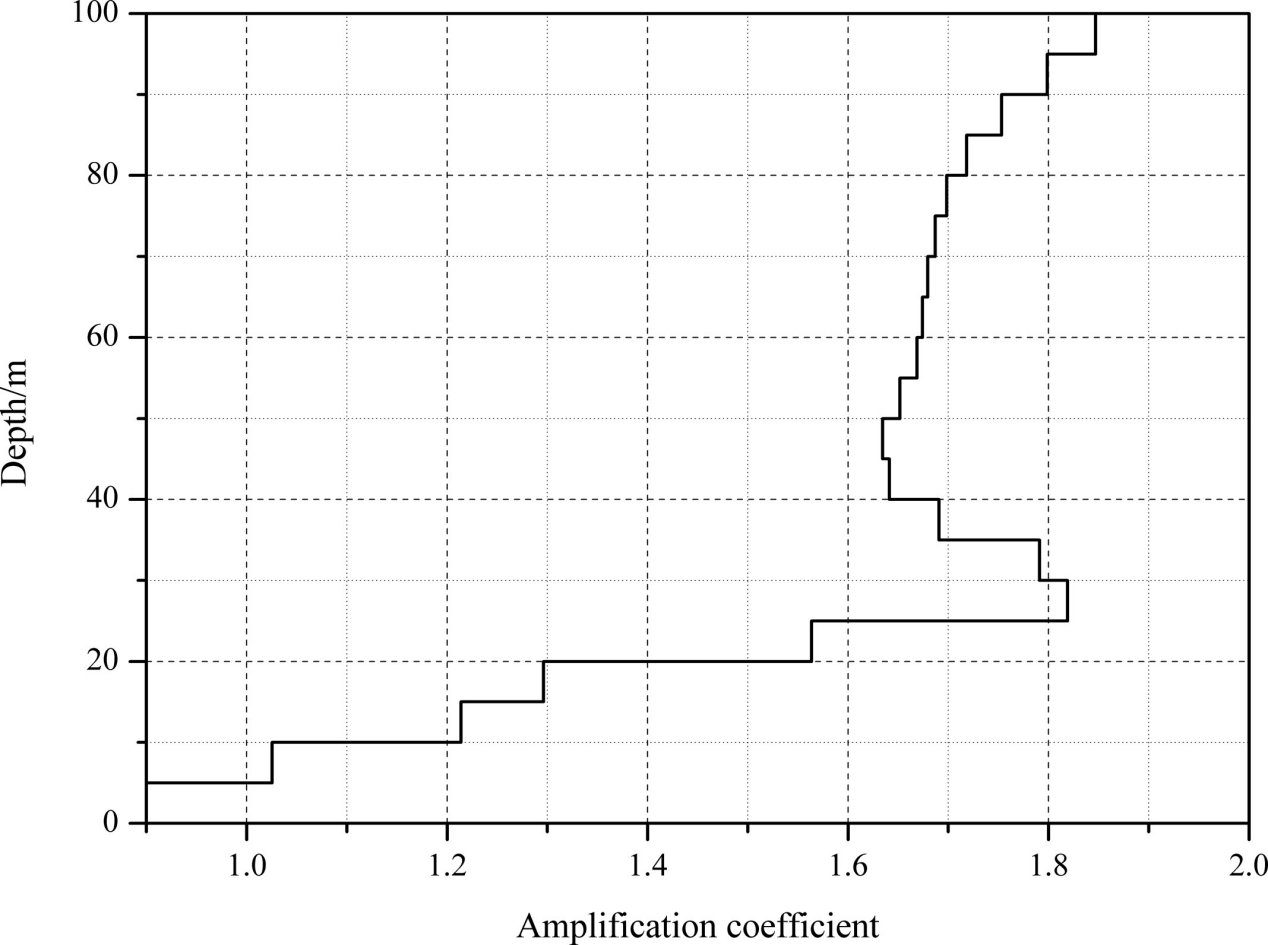
The distribution of the PGA amplification coefficients.

Fig 4 illustrates the ground motion acceleration time history at loess sites with different loess layer thicknesses. It can be intuitively seen that the phase difference of the time history under different soil conditions was relatively small, and the peak ground acceleration increased with the increasing loess layer thickness. To quantify the amplification effect at the loess site, the amplification coefficient, which is the ratio of peak ground acceleration (PGA) to input seismic acceleration, is introduced in this paper. The distribution of the PGA amplification coefficient is indicated in Fig 5. The dynamic response of the sites with different loess thicknesses reflected obvious amplification characteristics. The amplification coefficients rapidly increased with increasing soil layer thickness when the thickness is less than 30 meters. As the soil layer thickness further increased, the coefficients first decreased and then slowly increased. Thus, the nonlinear characteristics of the amplification effect at loess sites under seismic ground motion can be proven as well.

**Fig 6:**
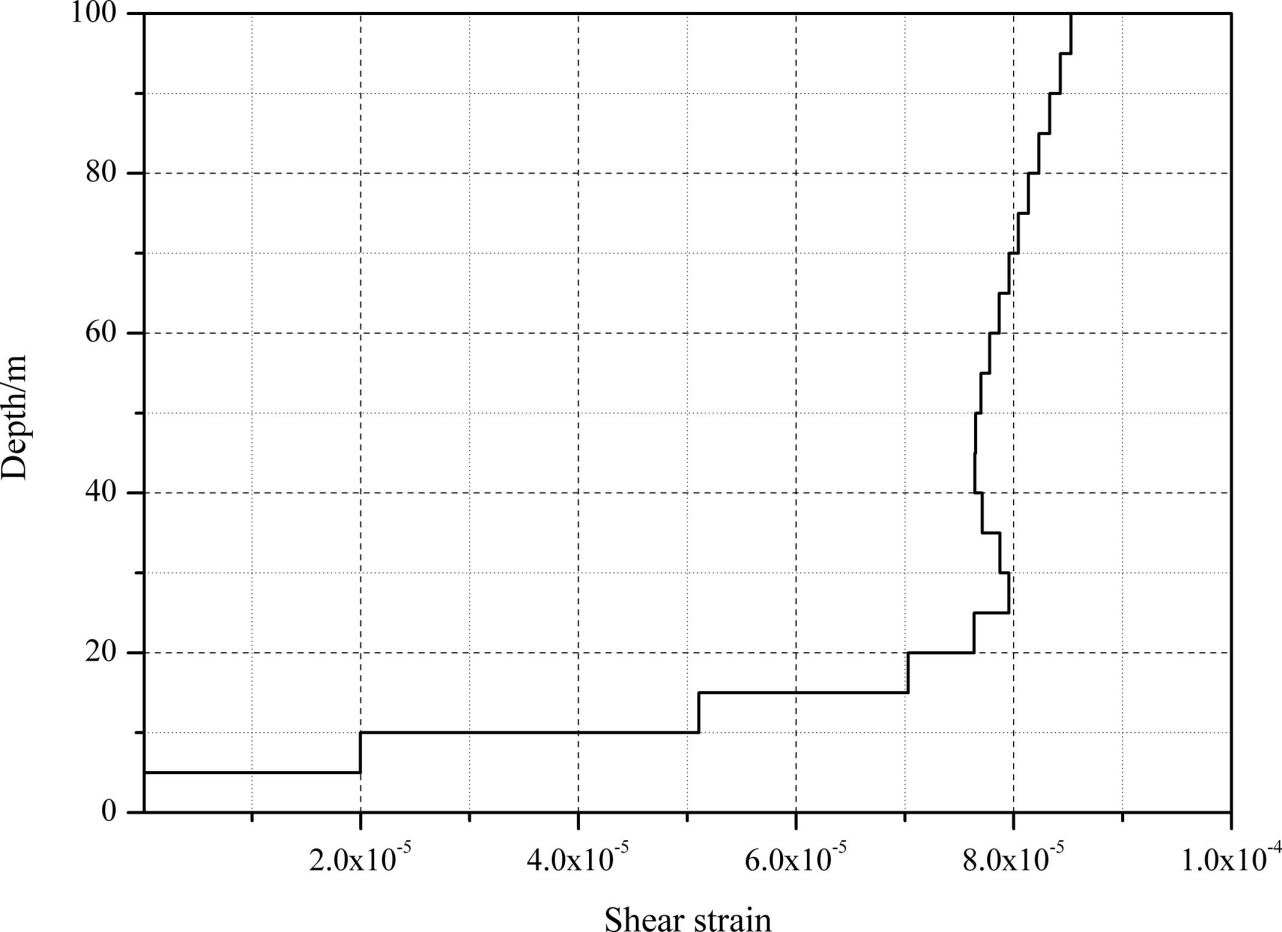
The distribution of the shear strain.

Fig 6 illustrates the distribution of loess maximum shear strains with different loess layer thicknesses under far-field earthquake excitations. The change in the maximum shear strains with loess layer thickness is analogous to the distribution of the PGA amplification coefficients. The minimum value of the average shear strain reached 2.4E^-5^, while the maximum value reached 8.6E^-5^ when the thickness of the overburden in the loess region was approximately 100 m.

**Fig 7:**
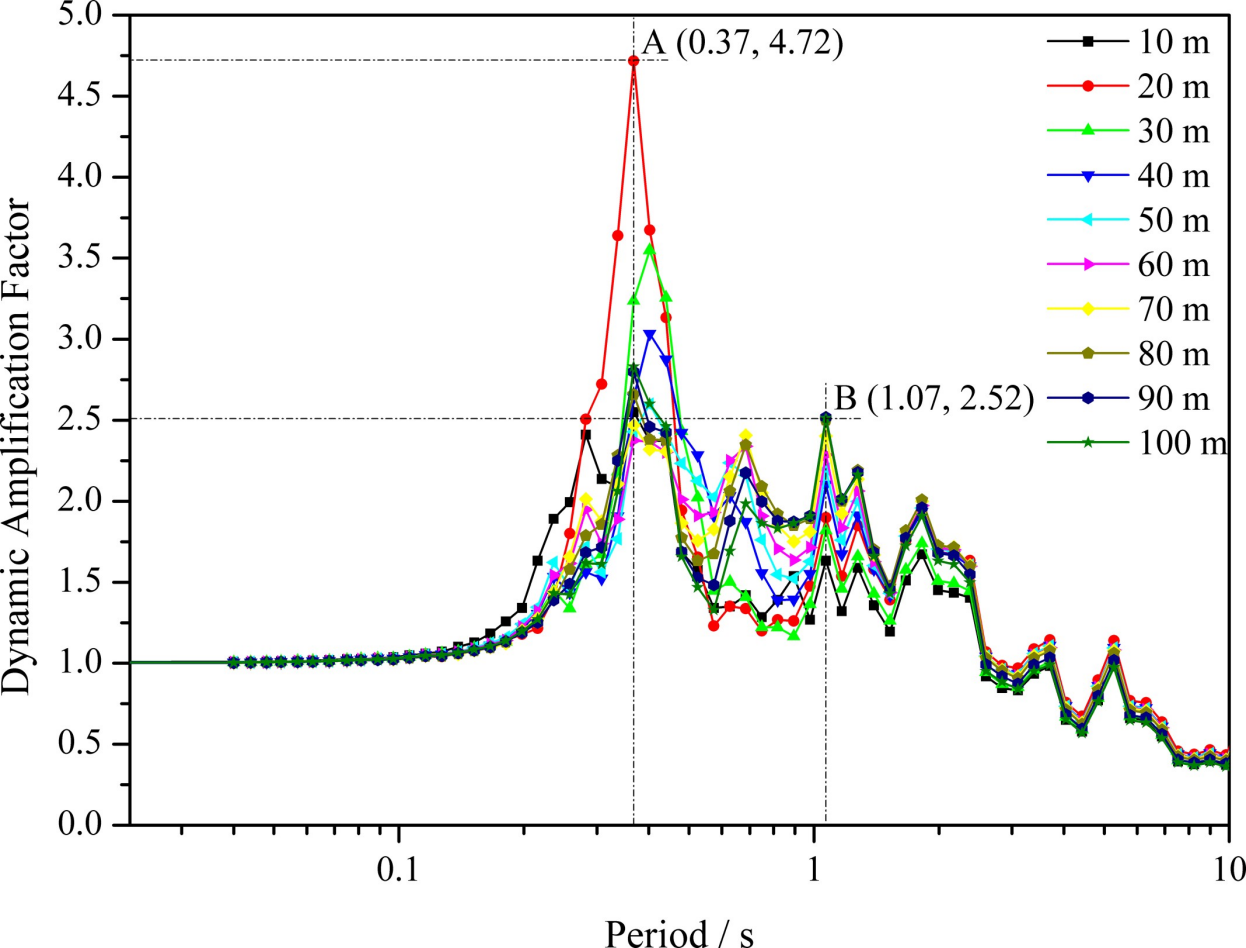
Acceleration spectrums under far field seismic ground motion.

The normalized acceleration response spectra of the loess site under far-field seismic ground motion are illustrated in Fig 7. It can be observed that the dynamic magnification factor of the short-period structures under far-field seismic ground motion range from 2.3 to 4.7. The point A indicate that the response is the strongest at the period of 0.37s when the depth of loess layer is 20 m. However, the values of the dynamic magnification factor of the long period (≥1s) structures range from 1.6 to 2.5. The point B indicate that the response is the strongest at the period of 1.07s when the depth of loess layer is 100 m.

### Slope topographic effects on ground motion

Through the above analysis and discussion, the amplification effects of soil sites on ground motions were presented. Therefore, on this basis, slope topographic effects on ground motion were studied by applying the nonlinear dynamic finite element analysis method, and the kinetic equation of the soil seismic response can be expressed as:

$$M\cdot \overset{¨}{x(t)}+C\cdot \dot{x(t)}+K\cdot x(t)=M\cdot \overset{¨}{x_{g}(t)}$$ (2)

where M, C and K are the mass matrix, stiffness matrix and damping matrix, respectively. $\overset{¨}{x_{g}(t)}$ refers to the bedrock ground motion acceleration.

When the ground motion is weak, the soil is in the stage of elastic deformation and its stress-strain relation follows Hook’s law. With the increase in ground motion acceleration, the soil starts to enter the yield stage. Then, the elastoplastic properties of soil need to be considered. For any stress increment, the total strain increment can be written as:

$$d\left\{\varepsilon \right\}=d\left\{\varepsilon^{e}\right\}+d\left\{\varepsilon^{p}\right\}={\left[D_{T}\right]}^{-1}d\left\{\sigma \right\}+d\lambda \left\{\frac{\partial F}{\partial \sigma }\right\}$$ (3)

where [*D_T_*] is the elastic matrix related to temperature and unfrozen water, {*ε*} is the strain, {*σ*} is the stress, {*ε^e^*} is elastic strain, {*ε^p^*} is plastic strain, dλ is the plastic coefficient, and F represents the plastic potential function.

In this paper, the Mohr-Coulomb elastic plastic yield criterion is used to express the plastic potential function. This model can be expressed as:

$$F=\frac{J_{1}}{3}\sin\varphi +\sqrt{J_{2}^{'}}(\cos\theta -\frac{1}{\sqrt{3}}\sin\theta \sin\varphi )-C\cos\varphi =0$$ (4)

where *J*_1_ is the first stress tensor invariant, $J_{2}^{'}$ denotes the second deviatoric stress tensor invariant, and *θ* is the lode angle. C denotes the cohesive strength, and *φ* denotes the internal friction angle.

### Numerical model and mechanical parameters

According to a survey of loess slopes in Lanzhou city, the characteristics of slope topography were determined. Then, the representative loess slope was selected for numerical analysis of the slope topographic effects, and a simplified finite element model was established.

**Fig 8:**
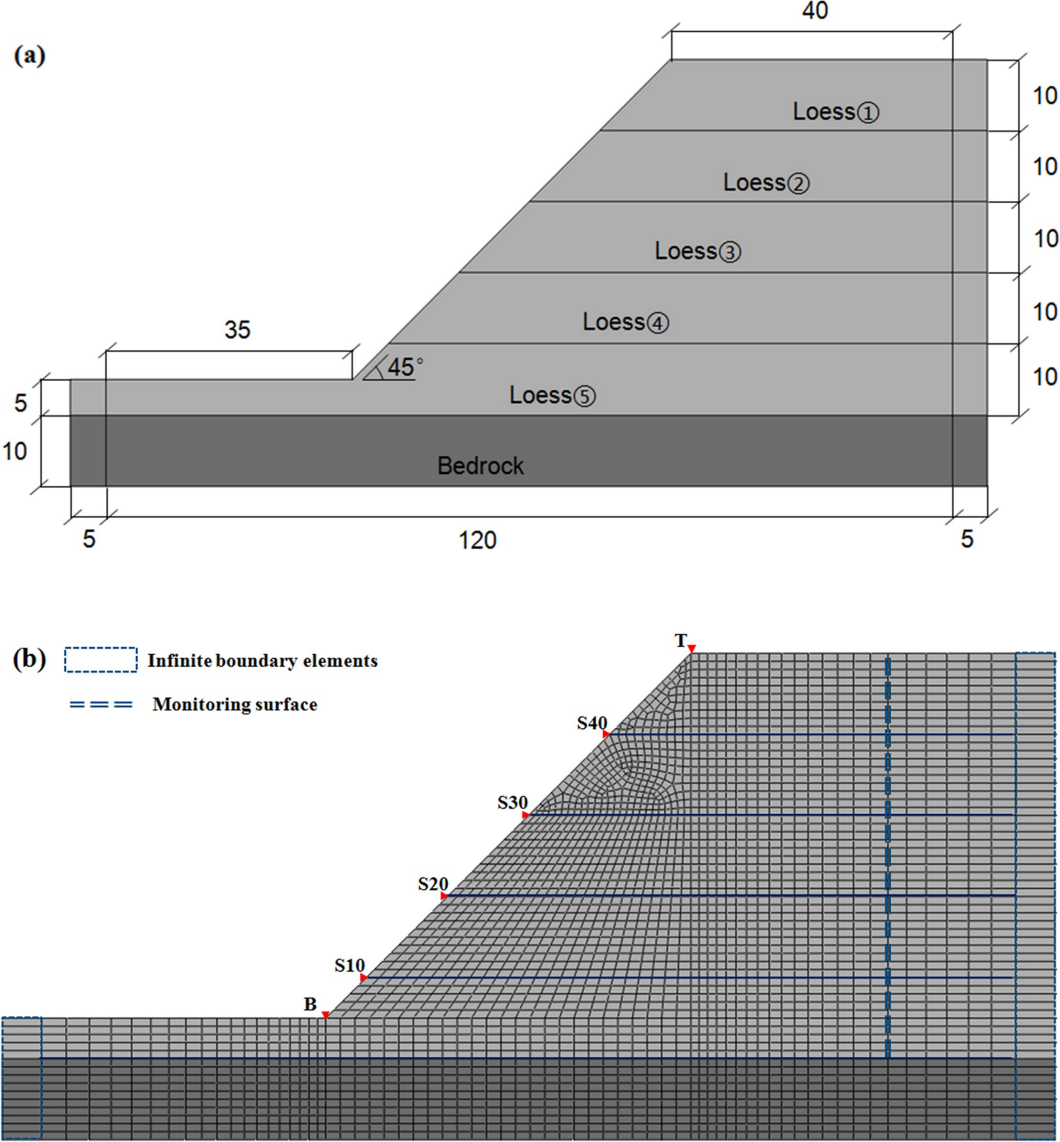
Models of finite element analysis: (a) Profile of the loess slope (unit: m); (b) Finite element model.

As shown in Fig 8A, the model of the loess slope comprised two layers: the upper loess layer and bottom bedrock layer. The loess was divided into five layers (ranging from ① to ⑤), and the thickness of each layer was set as 10 meters. The different densities and mechanical parameters, corresponding to the one-dimensional model, were endowed as well.

The finite element model of the loess slope is shown in Fig 8B. In this simulation, the slope angle was set to 45 degrees, and the numerical calculation was deduced based on the plane strain assumption. According to a study conducted by KUHLEMEYER, the grid size of the model should be less than 1/8−1/10 of the wavelength of input ground motion [23]. In this analysis, the computational model was meshed considering this influence, and the average size of the element was approximately 1 × 1 m.

To reduce the effect of boundary conditions on energy reflection and transmission properties, the coupling method of finite and infinite elements was utilized. On the left and right sides of the numerical model, an infinite element was used to simulate the actual situation, and thus, the stress wave reflection at the lateral boundary was prevented. Moreover, artificial boundary conditions were adopted at the bottom of the mode. The horizontal direction at the bottom was released, and the far-field seismic ground motion (Fig 1) was used as the input horizontal seismic wave for the numerical calculation. Considering the research purpose and significance, one monitoring surface away from the free surface of the slope was arranged inside the loess. Moreover, the observation point at the top of the slope and the slope toe observation point were arranged, as well as the points on the slope surface.

In dynamic finite element analysis, the soil is assumed to obey realistic elastoplastic and Mohr-Coulomb yield criteria [24]. The shear wave velocity (Vs) and mass density (ρ) are determined in a one–dimensional calculation; thus, the shear modulus (Gmax) of the soil can be calculated through the following relationship:

$$G\max=\rho V_{s}{}^{2}$$ (5)

In the formula mentioned above, the values of the shear modulus of the loess layer are different due to the variations in Vs at different depths. Moreover, the soil mechanical parameters for the finite element numerical calculation, which are shown in Table 1, were obtained by triaxial tests [25, 26].

**Table 1:** Soil mechanical parameters of the finite element model.

Lithology	Thickness (m)	Density (kN·m^-3^)	Shear modulus (kPa)	Poisson’s ratio	Cohesion (kPa)	Frictional angle (°)
Loess ①	10	1.60	6.40E+04	0.3	32	18
Loess ②	10	1.65	1.29E+05	0.3	35	18
Loess ③	10	1.70	1.97E+05	0.3	38	19
Loess ④	10	1.73	3.05E+05	0.3	42	20
Loess ⑤	10	1.76	4.06E+05	0.3	46	21
Bedrock	10	2.20	1.41E+06	0.2	120	34

Before dynamic calculation, the dynamic response characteristics of free field soil are evaluated using an equivalent-linear approach. Then, the dynamic shear modulus and damping ratio are determined using an iterative process. The initial shear modulus and damping ratio are given; subsequently, the shear strain is determined after the first calculation. Then new values related to the strain are obtained, and the next calculation is conducted. Finally, the ultimate shear modulus and damping ratio are established when the relative error of the shear strain is acceptable.

### Acceleration responses of loess slope

**Fig 9:**
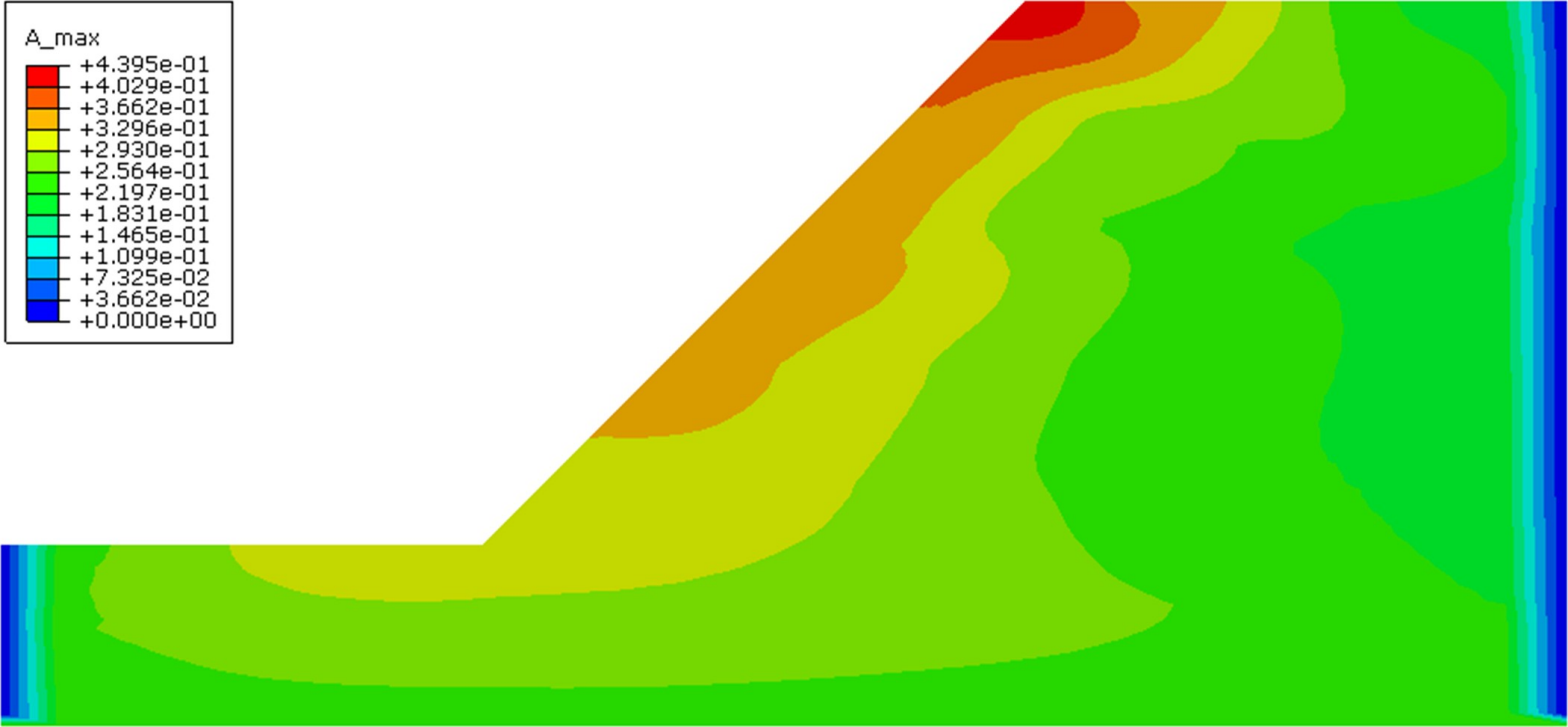
The horizontal acceleration contour map under the far-field seismic excitation (Unit: m.s^-2^).

It was discovered long ago that the slope topographic effects on ground motion exist under seismic excitation, which indicates acceleration amplifications (i.e., amplification of the ground acceleration values) along the slope. Fig 9 illustrates the horizontal acceleration contour map under far-field seismic excitation. It can be intuitively seen that the peak ground acceleration increases with increasing slope height, especially at the top slope position.

**Fig 10:**
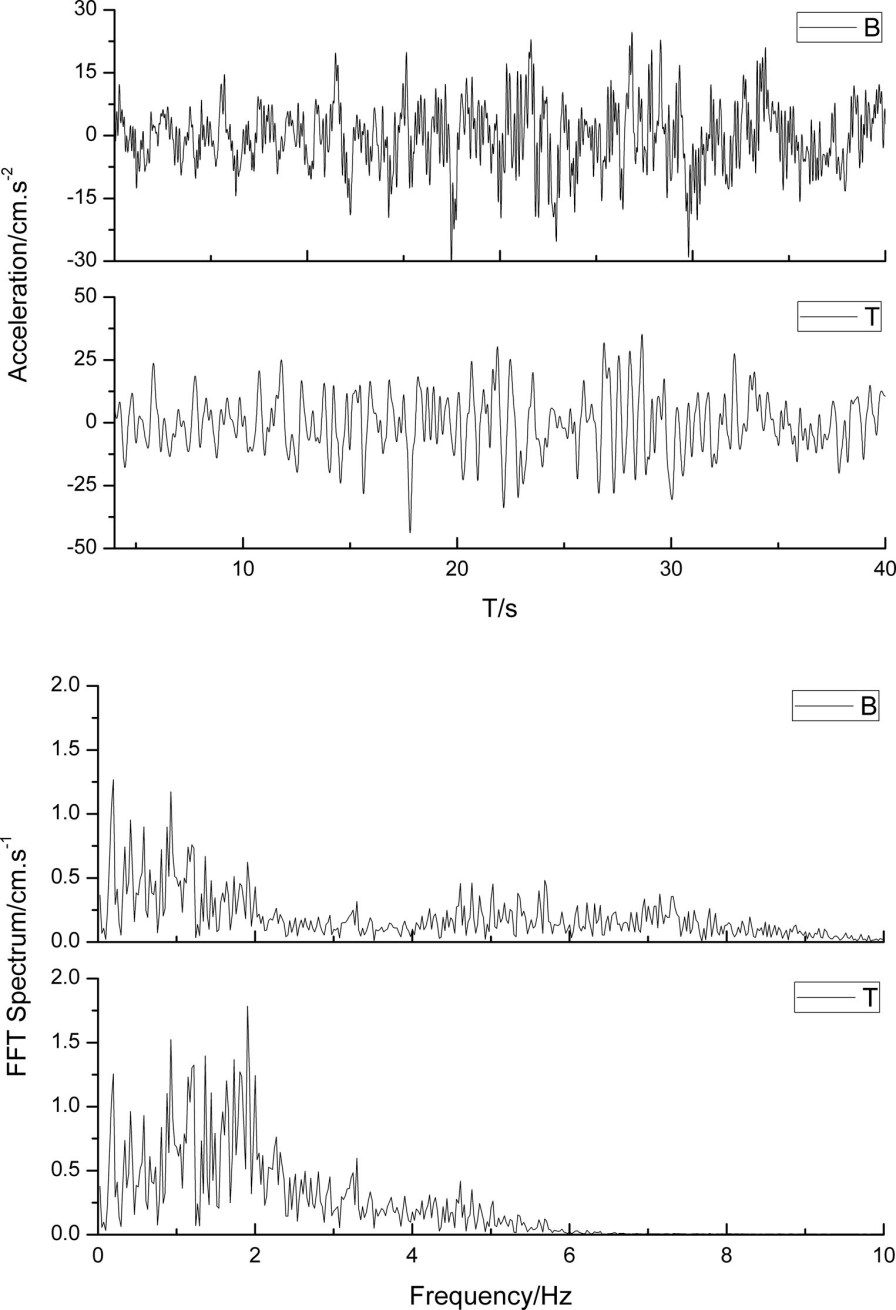
The acceleration time histories and the spectrum characteristics of different observation points. (a) the acceleration time histories; (b) the spectrum characteristics.

To quantify the amplification effect of the peak ground acceleration at the top slope position, the acceleration time histories of the observation points on the top of the slope and the slope toe observation point are extracted (Fig 10A). In addition, the spectrum characteristics are further discussed to determine the potential amplification mechanism (Fig 10B).

As shown in Fig 10, the peak ground acceleration of the position at the top of the slope (point T) can reach 44 cm.s^-2^, marking an 85% increase compared with the input peak ground acceleration, while the peak acceleration of the slope toe (point B) reaches 29 cm.s^-2^, marking an 30% increase. To validate this finite element model, the peak acceleration at different positions was compared with the PGA obtained from the seismic site response. Point T corresponds to the condition with a loess thickness of 50 m, while point B corresponds to a loess thickness of 5 m. Through this comparison, it is shown that the one-dimensional calculated results are very consistent with the finite element calculated results.

There are statistically significant differences in spectrum characteristics between the observation points on the top of the slope and slope toe. The ground motion on the top of the slope has an amplified low-frequency component, and the high-frequency components are suppressed. The dominant frequency of ground motion on the top of the slope is 1.9 Hz, while this values is below 1 Hz.

**Fig 11:**
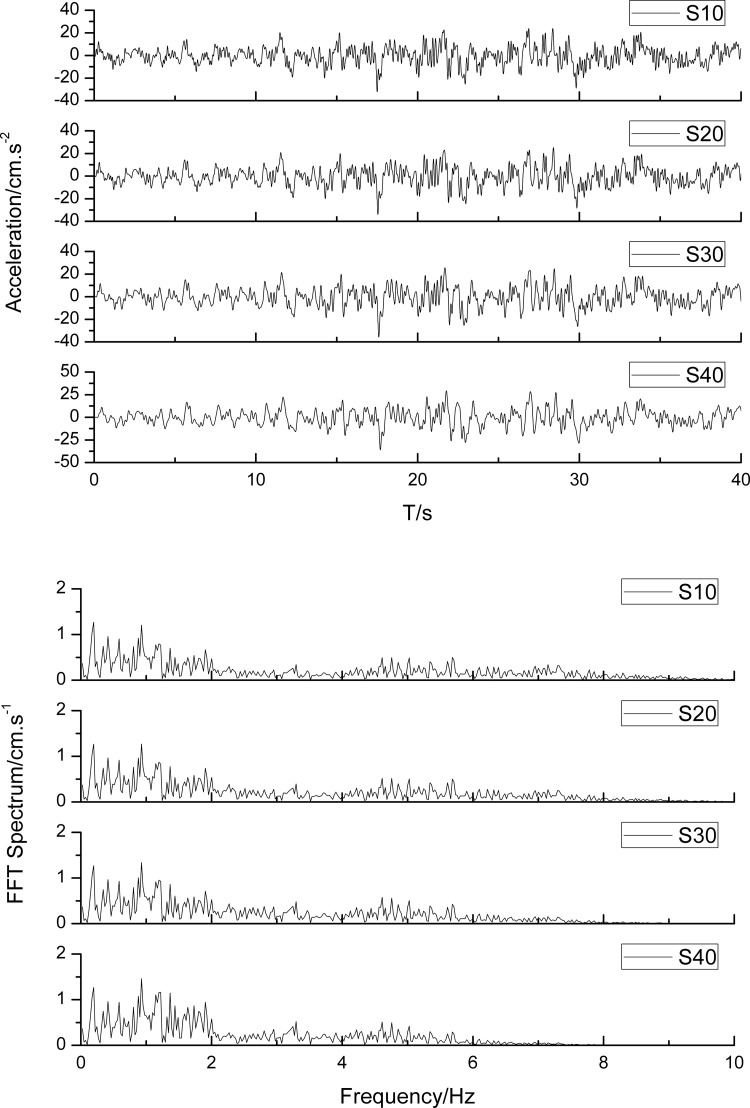
The acceleration time histories and the spectrum characteristics at the free surface. (a) the acceleration time histories; (b) the spectrum characteristics.

Fig 11 illustrates the acceleration time histories and the spectrum characteristics at the free surface with different slope heights. With seismic wave propagation along the slope, the high-frequency components of the ground motion are filtered out, while the low-frequency components remain unchanged. Moreover, the peak ground acceleration increases with increasing slope height.

**Fig 12:**
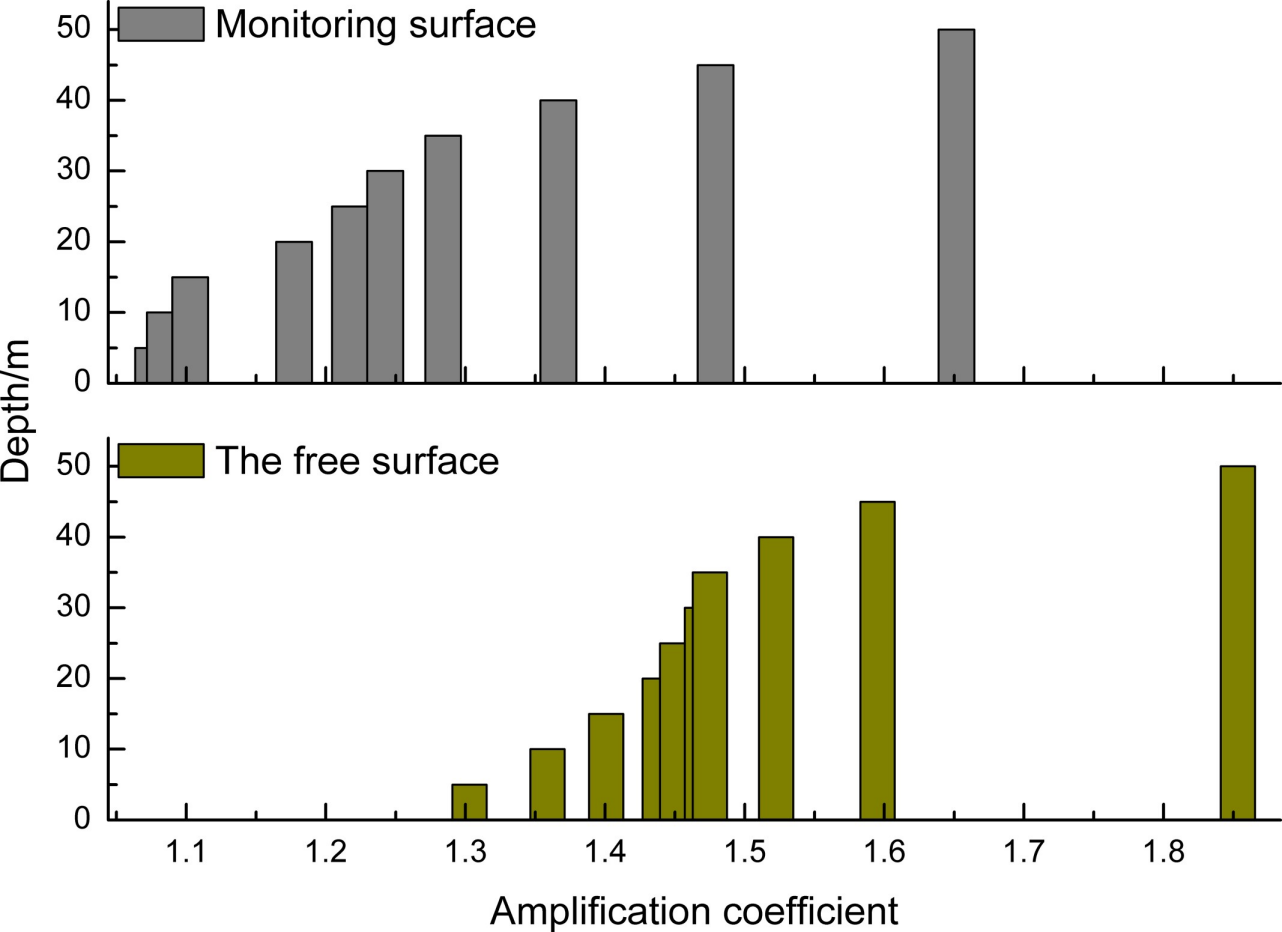
The distribution of the amplification coefficient of peak ground acceleration.

The distribution of the peak ground acceleration from slope toe observation point B to observation point T on the top of the slope, with different slope heights, is analyzed and compared with the loess amplification effect along the monitoring surface. Fig 12 illustrates the distribution of the amplification coefficient of peak ground acceleration at these two surfaces. The amplification coefficient of the observation point at the free surface is greater than that inside the soil at the same depth, and this difference is caused by the slope topography. The maximum amplification coefficient of the peak ground acceleration along the monitoring surface reaches 1.65, while this value can reach 1.85 along the free surface.

**Fig 13:**
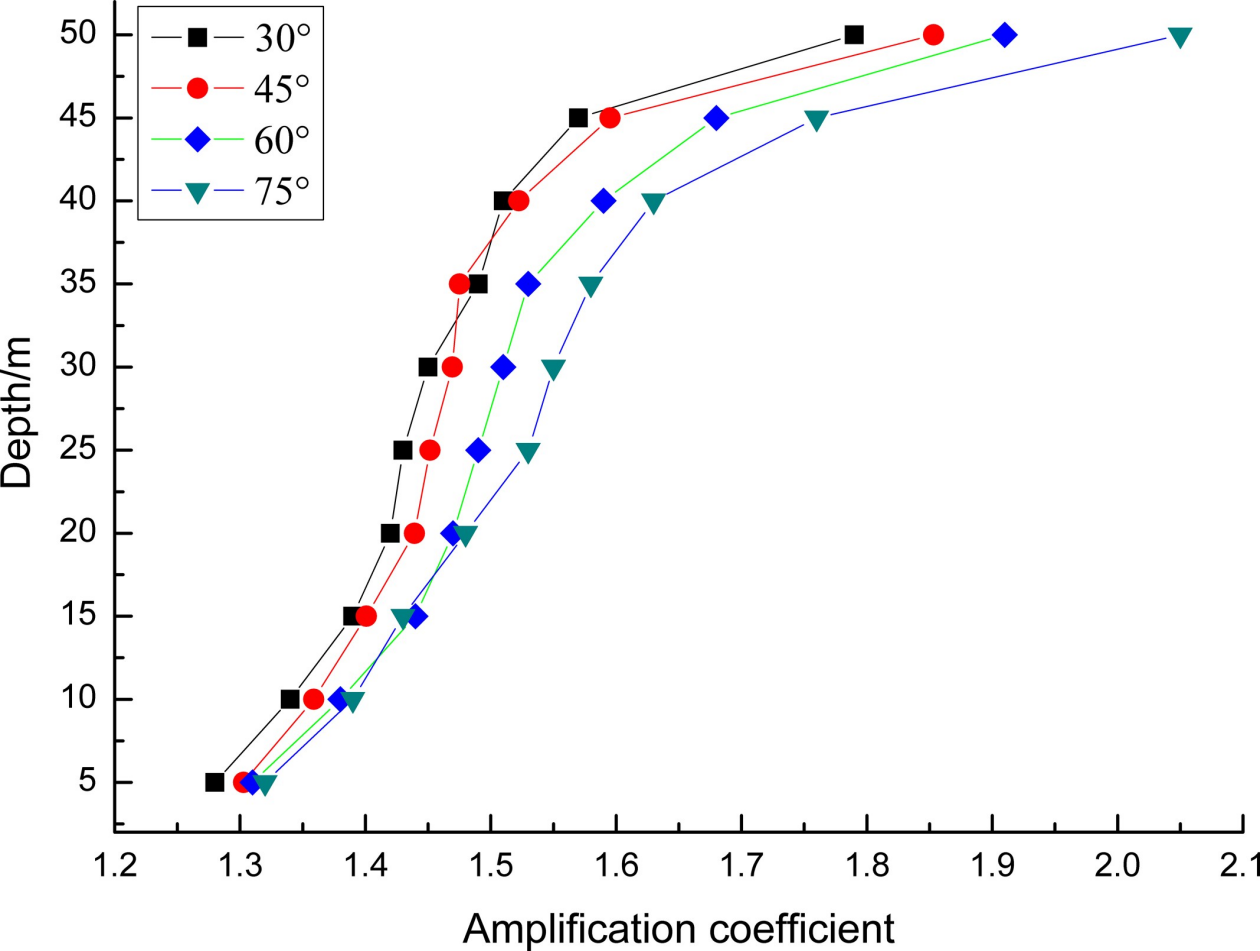
The impact of slope angle on the amplification coefficient of peak ground acceleration.

On this basis, the impact of the slope angle on the amplification coefficient of the peak ground acceleration is studied, which is shown in Fig 13. When the slope angle of the slope changes from 30° to 75°, the amplification coefficient of the peak ground acceleration ranges from 1.2 to 2.0. Moreover, the larger the slope angle is, the greater the amplification coefficient. It is further shown that the amplification of the peak ground acceleration mainly occurs in the upper slope, ranging in height from 40 to 50 meters.

## Discussion

Earthquake damage investigation in loess areas shows that the earthquake disaster characteristics are closely related to topography, lithology, geological structure, deposit thicknesses, etc. This paper mainly discusses the seismic response of loess sites with different deposit thicknesses and slope angles of topography, and the calculated results partly coincide with the field observation results. Numerical calculations considering more influential factors still need to be carried out. Moreover, the refinement analysis of the calculated results combined with the results of field observations after earthquakes in loess areas is the key research direction in the future.

The findings of this research indicate that the soil-filtering processes and selective amplification effects of seismic waves occur during propagation. The nonlinear amplification characteristics of the ground motion are mainly determined by the dynamic properties of the soil, especially the dynamic shear modulus and damping ratio. In addition, seismic site effects are strongly related to the seismic wave, such as near-field and far-field earthquake accelerograms, which have different frequency and amplitude characteristics.

As a complex factor, the slope topography has a significant effect on ground motion, reflecting that the dynamic acceleration response of the slope surface is greater than that in the slope. The peak ground acceleration of the position at the top of the slope is significantly increased. This phenomenon may be due to the reflection and refraction of seismic waves during wave propagation in the slope. Moreover, the resonance phenomenon may help explain the slope topographic effect. The dominant frequency of the site on the top of the slope becomes closer or equal to the main vibration frequency of the ground motion according to the in situ constant-fretting observation.

## Conclusions

In this work, numerical analysis of ground motion effects at loess sites under far-field bedrock ground motion was performed. Moreover, slope topographic effects on ground motion were studied. The following conclusions can be drawn from the results of this study:

1. The dynamic response of the sites with different loess thicknesses reflected amplification effects and had obvious nonlinear seismic response characteristics.
2. The values of the dynamic magnification factor of the short-period structures under far-field seismic ground motion ranged from 2.3 to 4.7, and this value of the long-period structures ranged from 1.6 to 2.5 in loess regions.
3. The amplification coefficient of the observation point at the free surface is greater than that of the point inside the soil at the same depth. The ground motion at the top of the slope has an amplified low-frequency component, and the high-frequency components are suppressed.
4. The amplification of the peak ground acceleration mainly occurs in the upper slope, and the larger the slope angle is, the greater the amplification coefficient.
5. In addition to the refraction and reflection theory of seismic waves, the resonance phenomenon may help explain the slope topographic effect through spectrum analysis.

## Supporting information

S1 Data (RAR)

## References

[1] Derbyshire E, Mellors T.W., 1988. Geological and geotechnical characteristics of some loess and loessic soils from China and Britain: a comparison. Engineering Geology. 25(2–4),135–175.

[2] Zhang Z Z. Forecast of seismic disasters in loess area. Beijing: Earthquake Press, 1999.

[3] Beck J L, Hall J F, 1986. Factors contributing to the catastrophe in Mexico City during the earthquake of September 19,1985. Geophys Res Lett. (13), 593~596.

[4] Boore D B, 2001. Comparisons of ground motions from the 1999 Chi-Chi earthquake with empirical predictions largely based on data from California. Bull. Seism. Soc. Am. (91), 1212~1217.

[5] Furumura T, Koketsu K, Wen K L, 2002. Parallel PSM/FDM hybrid simulation of ground motions from the 1999 Chi-Chi, Taiwan, earthquake. Pure Appl Geophys, 159, 2133~2146.

[6] Li X J, Zhou Z H, Yu H Y et al, 2008. Strong motion observations and recordings from the great Wenchuan earthquake. Earthquake Engineering and Engineering Vibration, (3): 235~246.

[7] Jousset P, Douglas J, 2007. Long-period earthquake ground displacements recorded on Guadeloupe (French Antilles). Earthquake Engng. Struct. Dyn, 36, 949~963.

[8] Koketsu K, Miyake H, 2008. A seismological overview of long-period ground motion. J Seismol, (12), 133~143.

[9] Maeda T and Sasatani T, 2008. Long-period ground motions from the 2003 Tokachi-oki earthquake. J Seismol, (12), 243~253.

[10] Cheng Y, Bai GL, 2017. Basic characteristic parameters and influencing factors of long-period ground motion records. Journal of Vibroengineering, 19(7), 5191–5207

[11] Arslan H, Siyahi B, 2010. Effect of nonlinearity on site response and ground motion due to earthquake excitation. Bulletin of Engineering Geology and the Environment, 69(2), 287–293

[12] Hartzell S, Meremonte M, Ramirez-Guzman L, McNamara D, 2014. Ground Motion in the Presence of Complex Topography: Earthquake and Ambient Noise Sources. Bulletin of the Seismological Society of America, 104(3), 451–466.

[13] Pandey B, Jakka RS, Kumar A, 2016. Influence of local site conditions on strong ground motion characteristics at Tarai region of Uttarakhand, India. Natural Hazards, 81(2), 1073–1089.

[14] Tran TT, Han SR, Kim D, 2018. Effect of probabilistic variation in soil properties and profile of site response. Soils and Foundations, 58(6), 1339–1349.

[15] Glinsky N, Bertrand E, Regniere J, 2019. Numerical simulation of topographical and geological site effects. Applications to canonical topographies and Rognes hill, South East France. Soil Dynamics and Earthquake Engineering, 116, 620–636. doi: 10.1016/j.soildyn.2018.10.020

[16] Wu Z. J, Wang L.M, Wang P, 2013. Influence of site conditions on ground motion at far field loess sites during strong earthquake. Journal of Central South University, (20), 2333–2341.

[17] Wang L.M, Wu Z.J, 2010. Influence of site condition on seismic amplification effects during the Wenchuan earthquake. Journal of Civil, Architectural & Environmental Engineering, 32 (S2): 175–178.

[18] Shi Y.C, Wang L.M, Zhang Y, 1999. Effects of overburden thickness and topography of loessial sites on earthquake ground motion, Northwestern Seismological Journal, 21 (2): 203–208.

[19] Jonathan P Stewart, Shyh-Jeng Chiou, Jonathan D Bray, et al, 2002. Ground motion evaluation procedures for performance-based design. Soil Dynamics and Earthquake Engineering, 22, 765–772. doi: 10.1016/S0267-7261(02)00097-0

[20] Iwan, W. D, 1994. Important Near-Field Ground Motion Data from the Landers Earthquake. Proceedings, 10th European Conference on Earthquake Engineering, Vienna, Austria, 229–234.

[21] Li X.J., 1989. The Soil Seismic Response of One-dimensional Linear Calculation Program, Seismic Microzonation (Theory and Pratice). Earthquake Publishing House, Beijing, 250–265.

[22] Wang L. M, Wu Z. J, Xia K, 2017. Effects of site conditions on earthquake ground motion and their applications in seismic design in loess region. Journal of Mountain Science, 14(6),1185–1193.

[23] KUHLEMEYER R L, LYSMER J, 1973. Finite element method accuracy for wave propagation problems. Journal of Soil Mechanics and Foundation Division, 99(5): 421–427.

[24] Che A. L, Iwatate T., Ge X, R, 2006. Dynamic behaviors of subway structure subjected to strong earthquake motions using shaking table tests and dynamic analyses. Rock and Soil Mechanics, 27(8), 1293–1298.

[25] Wang Z.J, Luo Y.S, Wang R.R, Yang L.G, Tan D.Y, 2010. Experimental study on dynamic shear modulus and damping ratio of undisturbed loess in different regions. Chinese Journal of Geotechnical Engineering, 32(9), 1464–1469.

[26] Song B. H, Tsinaris A, Anastasiadis A, Pitilakis K, Chen W.W, 2017. Small-strain stiffness and damping of Lanzhou loess. Soil Dynamic and Earthquake Engineering, 95, 96–105.

